# Testicular involution prior to sex change in gilthead seabream is characterized by a decrease in DMRT1 gene expression and by massive leukocyte infiltration

**DOI:** 10.1186/1477-7827-5-20

**Published:** 2007-06-04

**Authors:** Sergio Liarte, Elena Chaves-Pozo, Alicia García-Alcazar, Victoriano Mulero, José Meseguer, Alfonsa García-Ayala

**Affiliations:** 1 Department of Cell Biology, Faculty of Biology, University of Murcia, Campus Universitario de Espinardo, 30100 Murcia, Spain; 2 Oceanographic Centre of Murcia, Spanish Oceanographic Institute (IEO), Carretera de la Azohía s/n. Puerto de Mazarrón, 30860 Murcia, Spain

## Abstract

**Background:**

Leukocytes are found within the testis of most, if not all, mammals and are involved in immunological surveillance, physiological regulation and tissue remodelling. The testis of seasonal breeding fish undergoes a regression process. In the present study, the second reproductive cycle (RC) of the protandrous seasonal teleost fish, gilthead seabream, was investigated and the presence of leukocytes analysed. Special attention has been paid to the testicular degenerative process which is particularly active in the last stage of the second RC probably due to the immediacy of the sex change process.

**Methods:**

Sexually mature specimens (n = 10–18 fish/month) were sampled during the second RC. Some specimens were intraperitoneally injected with bromodeoxyuridin (BrdU) before sampling. Light and electron microscopy was used to determine the different stages of gonadal development and the presence of leukocytes and PCR was used to analyse the gene expression of a testis-differentiating gene and of specific markers for macrophages and B and T lymphocytes. Immunocytochemistry and flow cytometry were performed using a specific antibody against acidophilic granulocytes from the gilthead seabream. Cell proliferation was detected by immunocytochemistry using an anti-BrdU antibody and apoptotic cells by in situ detection of DNA fragmentation.

**Results:**

The fish in the western Mediterranean area developed as males during the first two RCs. The testis of all the specimens during the second RC underwent a degenerative process, which started at post-spawning and was enhanced during the testicular involution stage, when vitellogenic oocytes appeared in the ovary accompanied by a progressive increase in the ovarian index. However, only 40% of specimens were females in the third RC. Leukocytes (acidophilic granulocytes, macrophages and lymphocytes) were present in the gonad and acidophilic granulocyte infiltration occurred during the last two stages. At the same time DMRT1 gene expression decreased.

**Conclusions:**

The results demonstrate that innate and adaptive immune cells are present in the gonads of gilthead seabream. Moreover, the whole fish population underwent a testicular degenerative process prior to sex change, characterized by high rates of apoptosis and necrosis and accompanied by an infiltration of acidophilic granulocytes and a decrease in DMRT1 levels.

## Background

The testis is a dynamic tissue that is tightly controlled not only by hormones but also by local control mechanisms in which cell to cell interactions are involved. Leukocytes (macrophages, lymphocytes and mast cells) are found within the testes of most, if not all, mammals and are involved in immunological surveillance, physiological regulation and tissue remodelling [1-4]. Although the major focus of gonadal leukocyte research has been mammals, studies in other vertebrates may shed some light on the evolutionary mechanisms involved in the dysregulation of normal gonad physiology. Moreover, fish represent an attractive group of organisms for studying sex determination from the evolutionary point of view because they cover the complete range of sexuality, from hermaphroditism to gonochorism [5]. However, most of the fish models used to analyze the genes involved in sex determination and differentiation are gonochorism [6]. Unlike in mammals, sex-determining genes have not been described in fish, although some candidates have been proposed [6]. Thus, based on evolutionary conservation, it has been suggested that DMRT1 (double sex-and mab3-related transcription factor 1) may be involved in sex differentiation from invertebrates to human [6,7]. In trout, for example, DMRT1 has been described as being important in male differentiation but not in female differentiation. Moreover, its expression can be regulated by hormonal treatments that usually succeed in producing phenotypical sex change [8].

The gilthead seabream (*Sparus aurata *L.) is a protandrous hermaphroditic sparid fish with a heterosexual gonad that undergoes sex change during the second or third year of life, depending on the natural environment of the populations studied [9-11]. In most Mediterranean areas, the specimens undergo this sex change during the second year of life [12]. Several studies have dealt with the gilthead seabream sex change and its female physiology [13,14], but few studies have followed the male physiology throughout the reproductive cycle (RC). Our previous studies on the first RC of the gilthead seabream demonstrated that acidophilic granulocytes (produced in the head-kidney, the equivalent to mammalian bone marrow) infiltrate the testis under endocrine and paracrine regulation, display tissue specific functions and are involved in the testis degeneration that takes place during post-spawning [15-18].

The aim of this study was to characterize the second RC, prior to sex change, of the gilthead seabream, focusing on cell renewal (proliferation, apoptosis and necrosis) and the presence of acidophilic granulocytes, macrophages and T and B lymphocytes in the testicular and ovarian area of the gonad. Moreover, since in the heterosexual gonad of sparids the mechanisms involved in the differentiation of one sex and those which block the development of the other might coexist, a study of the testis differentiating gene, DMRT1, in the gonads of gilthead seabream throughout the second RC was thought to be of interest.

## Methods

### Fish

Healthy specimens of sexually mature male gilthead seabream *Sparus aurata *L. (Sparidae, Perciform, Teleostei), with a body weight (bw) of 100 g, were obtained in November 2004, from CULMAMUR, S.L. (Águilas, Spain). The fish were kept at the Spanish Oceanographic Institute (Mazarrón, Murcia), in 14 m^3 ^running seawater aquaria (dissolved oxygen 6 ppm, flow rate 20% aquarium volume/hour) with natural temperature and photoperiod, and fed twice a day with a commercial pellet diet (Trouvit, Burgos, Spain). Fish were fasted for 24 h before sampling. The fish with bw ranging from 230 to 1020 g were sampled from October 2005 to October 2006 (n = 10–18 fish/month). In order to determine the final sex ratio of the population, a final sampling was performed in November 2006 (n = 30 fish). At all sampling times the specimens were weighed, and the gonads and the head-kidneys were removed. Gonads were weighed and processed for light and electron microscopy, flow cytometry and gene expression studies, as described below. The head-kidneys were used as positive control in flow cytometry assays. Some specimens (n = 5/month) were weighed and injected intraperitoneally (i.p.) with 50 mg/kg bw of 5-bromo-2'-deoxyuridine (BrdU, Sigma) 2 h before sampling.

The experiments described comply with the Guidelines of the European Union Council (86/609/EU) and the Bioethical Committee of the University of Murcia (Spain) for the use of laboratory animals.

### Light microscopy and immunocytochemical staining

The gonads were fixed in Bouin's solution or 4% paraformaldehyde solution, embedded in paraffin (Paraplast Plus; Sherwood Medical) and sectioned at 5μm. Some sections were stained with hematoxylin-eosin in order to determine the reproductive stage and the degree of development of each fish, whereas others were subjected to an indirect immunocytochemical method [19] using a monoclonal antibody (mAb) specific to gilthead seabream acidophilic granulocytes (G7) [20] and an anti-BrdU mAb (Caltag) to determine the presence of acidophilic granulocytes and proliferative cells, respectively, as has been previously described [16].

The sections were slightly counterstained with Maller hematoxylin. The specificity of the reactions was determined by omitting the first antiserum and in the case of BrdU detection, using gonad sections from fish that had not been injected with BrdU. Slides were examined with an Axiolab (Zeiss) light microscope.

### In situ detection of DNA fragmentation (TUNEL)

TUNEL was performed to identify apoptotic cells (*in situ *cell death detection kit; Roche), as described previously [18]. Slides were examined with an Axiolab (Zeiss) light microscope.

### Electron microscopy

Samples were fixed with 4% glutaraldehyde in 0.1 M cacodylate buffer (pH 7.2) for 4–5 h at 4°C, postfixed in 1% osmium tetroxide in 0.1 M cacodylate buffer for 1 h at 4°C, and then embedded in Epoxi resins. Ultrathin sections were obtained with a Reichert-Jung ultramicrotome, contrasted with uranyl acetate and lead citrate, and examined with a Zeiss EM 10C electron microscope.

### Cell suspensions

The gonad and head-kidney cell suspensions were obtained as described previously [15].

### Flow cytometry

Aliquots of 5 × 10^6 ^cells were washed in flow cytometry (FC) buffer [PBS containing 2% fetal calf serum (FCS) and 0.05% sodium azide] and incubated for 30 min on ice with 100μl of G7, at the optimal dilution of 1:100 in FC buffer. After being washed, cell suspensions were incubated for 30 min on ice with 50μl of fluorescein isothiocyanate (FITC) labelled anti-mouse F(ab')_2 _fragments of goat antibody (Caltag) at the optimal dilution of 1:1000 in FC buffer. Cells were then washed twice and data were collected in the form of two parameter forward-scatter (FSC) and side-scatter (SSC) dot plots and green fluorescence (FL1) histograms by using a fluorescence-activated cell sorter (Becton Dickinson). Each G7 staining was carried out in duplicate.

### Analysis of gene expression

Total RNA was extracted from gonad fragments (n = 4–5 gonads/month) with TRIzol Reagent (Invitrogen) following the manufacturer's instructions and treated with DNase I, amplification grade (1 unit/μg RNA, Invitrogen). The SuperScript III RNase H^- ^Reverse Transcriptase (Invitrogen) was used to synthesize first strand cDNA with oligo-dT_18 _primer from 1μg of total RNA, at 50°C for 60 min. Total mRNA were obtained after mixing the same amount of mRNA from 4–5 fish/month.

The mRNA levels of the testis differentiating gene, DMRT1, were analyzed by real-time PCR with an ABI PRISM 7700 instrument (Applied Biosystems) using SYBR Green PCR Core Reagents (Applied Biosystems). Reaction mixtures were incubated for 10 min at 95°C, followed by 40 cycles of 15s at 95°C, 1 min at 60°C, and finally 15s at 95°C, 1 min 60°C and, 15s at 95°C. For each mRNA, gene expression was corrected by the ribosomal protein S18 content in each sample, and in all cases, each PCR was performed with triplicate samples. The primers used are shown in Table 1.

**Table 1:** Primers used for gene expression analysis by RT-PCR. Gene name abbreviation, accession number, primer sequence (forward and reverse) and annealing temperature used for gene expression analysis.

**Gene**	**Accession Number**	**Annealing temperature**	**Name**	**Sequence (5'-3')**
β-actin	X89920	55	F	ATCGTGGGGCGCCCCAGGCAC
β-actin			R	CTCCTTAATGTCACGCACGATT
S18	AM490061	60	F	AGGGTGTTGGCAGACGTTAC
S18			R	CTTCTGCCTGTTGAGGAACC
DMRT1	AM493678	60	F	GATGGACAATCCCTGACACC
DMRT1			R	GGGTAGCGTGAAGGTTGGTA
M-CSFR	AM050293	60	F3	CTGCCCTACAATGACAAG
M-CSFR			R4	TCAGACATCAGAGCTTCC
TCR-β	AM490435	60	F1	GCTTCTTCAATGGGACAGGA
TCR-β			R1	CCGTAGACACAGCCCTTGAT
IgM-H	AM493677	60	F1	CAGCCTCGAGAAGTGGAAAC
IgM-H			R1	GAGGTTGACCAGGTTGGTGT

The mRNA levels of macrophage colony stimulating factor receptor (M-CSFR), T cell receptor β chain (TCR-β) and immunoglobulin M heavy chain (IgM-H) genes, as markers for macrophages and T and B lymphocytes, respectively, were analyzed by semi-quantitative PCR with an Eppendorf Mastercycle Gradient Instrument (Eppendorf). Reaction mixtures were incubated for 2 min at 95°C, followed by 35 cycles of 45s at 95°C, 45s at the specific annealing temperature for each gene (see Table 1), 1 min at 72°C, and finally 10 min at 72°C. As a RT-PCR control expression *β-actin *was used.

### Analysis of the reproductive stage

As an index of the reproductive stage, we calculated the gonadosomatic index (GSI) as 100 × [W_G_/W_B_] (%), where W_G _is gonad weight (in grams) and W_B _is body weight (in grams).

As an index of ovarian development, the ovarian ratio, calculated as ovarian area (mm^2^)/total gonad area (mm^2^) × 100 (%) was measured, taking longitudinal sections (n = 5–14) stained with hematoxilin-eosin from the middle part of the gonad (n = 3/month) and in all cases corresponding to approximately 30% of the total volume of the organ. The ovarian area included the ovigerous lamellae and the ovarian cavity, and was drawn manually over the digital image. The total area of the gonad covered the ovarian area, the spermatogenetic tubules and the efferent duct, and was measured using an image analysis threshold method employed to differentiate borders. The ratio between these two areas was calculated from measurements of gonad tissue images obtained with an Olympus SZ11 overhead projector, a Sony DXC 151 AP video camera, and the software MIP 4.5 Consulting Image Digital (CID, Barcelona).

In order to determine oocyte growth, oocyte nuclear and cell diameters were drawn manually and measured by image analysis using an Axiolab (Zeiss) light microscope, a CoolSNAP digital camera (RS Photometrics) and SPOT Advance 3.3 software (Diagnostic Instruments, Inc.).

### Calculations and statistics

FC assays were performed with cells from at least three different fish. A quantitative study of the FC results was made by using the statistical option of the Lysis Software Package (Becton Dickinson). The number of oocytes measured (n = 111–269) was always higher than the number obtained by the formula (standard deviation · 0.83/mean · 0.05)^2^. All data were analyzed by ANOVA and a Waller-Duncan multiple range test to determine differences among groups (P ≤ 0.05).

## Results

### Morphology, cell proliferation and apoptosis in the testicular area of the gonad

**Figure 1:**
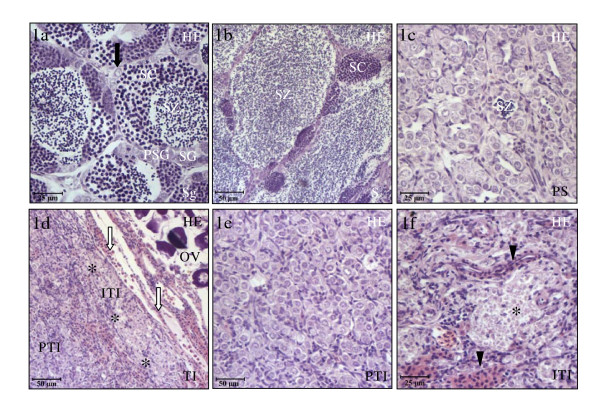
**Testicular area of the gonad**. The testicular area of the gonad at different stages of the second RC stained with hematoxylin-eosin. At spermatogenesis (**a**), spermatogonia stem cells and all germ cell type cysts formed the tubules of the testis. At spawning (**b**), the tubules are larger and full of free spermatozoa. At post-spawning (**c**), the main cell types in the tubules are spermatogonia stem cells and primary spermatogonia cysts. Some remaining spermatozoa can also be seen. At testicular involution (**d-f**), the testis is formed by spermatogonia stem cells and primary spermatogonia cysts that compose a dense tissue with no lumina in the tubules. Two morphological areas can be distinguished: the peripheral testicular area (**d,e**) and the internal testicular (**d,f**) area which is close to the efferent duct and the ovarian area and presents large necrotic areas surrounded by eosinophilic granulated cells. Scale bar = 25 μm (**a,c,f**,) and 50μm (**b,d,e**). Sg, Spermatogenesis; S, spawning; PS, post-spawning; TI, testicular involution; SG, spermatogonia cysts; PSG, primary spermatogonia cysts; SC, spermatocytes cysts; SZ, spermatozoa; PTI, peripheral testicular area in the involution stage; ITI, internal testicular area in the involution stage; OV, ovarian area; (arrow), spermatogonia stem cell; (arrow heads), eosinophilic cells; (white arrows), efferent duct; (asterisk), necrotic areas.

All the specimens during the second RC were male. The testicular area was composed of tubules consisting of spermatogonia stem cells and cysts (a cohort of synchronically developed germ cells enclosed by a cohort of Sertoli cells) of primary spermatogonia, A and B spermatogonia, spermatocyte, and spermatids and free spermatozoa. Based on the morphological changes observed in the testicular area, the second RC can be divided into four stages: spermatogenesis, spawning, post-spawning and testicular involution. During spermatogenesis (from October to January, Fig. 1a), spawning (February, Fig. 1b) and post-spawning (March, Fig. 1c) the testis showed a similar morphology to that described in the first RC of the gilthead seabream [16]. Interestingly, after post-spawning, during the testicular involutive stage (from April to July) the involutive process which started at post-spawning became more apparent. As regards morphology during the testicular involution stage, the testicular area could be divided into two areas (Fig. 1d): (i) the testicular peripheral area located at the edge of the gonad and formed by a dense tissue with no tubular lumen and a germinal compartment composed of spermatogonia stem cells and some primary spermatogonia cysts, similar to that observed at post-spawning (Fig. 1d,e), and (ii) the testicular internal area located next to the efferent duct and the ovary and formed by wide necrotic areas (Fig. 1d, f) composed of cell debris and surrounded by well developed interstitial tissue with large clusters of eosinophilic cells (Fig. 1f).

**Figure 2:**
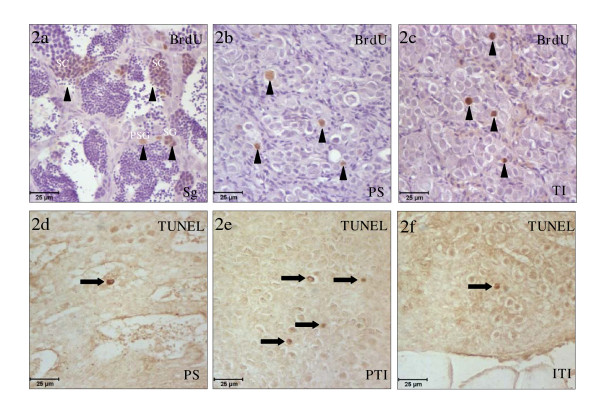
**Cell proliferation and apoptosis in the testicular area of the gonad**. Testicular area of the gonad at different stages of the second RC immunostained with the anti-BrdU mAb (**a-c**) or labeled with TUNEL (**d-f**). At spermatogenesis (**a**), spermatogonia stem cells and primary spermatogonia and spermatocytes cysts proliferate. At post-spawning (**b**) and testicular involution (**c**) spermatogonia stem cells and primary spermatogonia cysts were inmunostained. Only at post-spawning (**d**) and testicular involution (**e,f**) were apoptotic cells observed. Notice that in the testicular peripheral area (**e**) the number of TUNEL positive cells is higher than in the testicular internal area (**f**). Scale bar = 25 μm (**a-f**). (arrowheads), proliferative cells; (arrows), TUNEL positive cells; Sg, Spermatogenesis; PS, post-spawning; TI, testicular involution; PTI, peripheral testicular area in the involution stage; ITI, internal testicular area in the involution stage.

The immunodetection of BrdU and the *in situ *detection of DNA fragmentation, in the testicular area, were associated with the second RC (Fig. 2). Thus, the proliferative cell types and their proliferation rates during spermatogenesis (Fig. 2a), spawning (data not shown) and post-spawning (Fig. 2b) were similar to that observed during the same stages of the first RC [16]. During testicular involution (Fig. 2c) many BrdU positive spermatogonia stem cells and primary spermatogonia cysts could be seen randomly distributed throughout the testis.

Apoptosis is one of the most important mechanisms of cell death and is involved in several physiological processes related with tissue renewal. In the testicular area of the gonad, apoptosis was only detected during post-spawning (Fig. 2d) and testicular involution (Fig. 2e, f). Surprisingly, the apoptotic cells in the peripheral testicular area (Fig. 2e) were more numerous than in the internal testicular area during the testicular involution stage (Fig. 2f). In both stages, apoptotic cells had the features of primary spermatogonia, that is, they were set in the germinal compartment, isolated from each other, and possessed large and round nuclei.

### Morphology, cell proliferation and apoptosis in the ovarian area of the gonad

**Figure 3:**
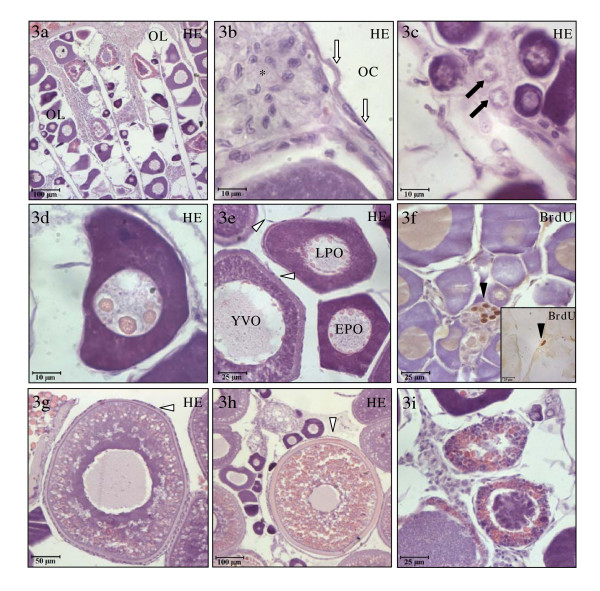
**The ovarian area of the gonad**. Ovarian area of the gonad at different stages of the second RC stained with hematoxylin-eosin (**a-e,g-i**) and immunostained with BrdU mAb (**f, inset f**). The ovarian epithelium forms longitudinal lamellae that extend into the central ovarian cavity (**a**). A squamous epithelium lines these lamellae at the luminal surface, below which a group of undifferentiated somatic cells were observed (**b**). The ovarian lamellae are formed by nests of oogonia (**c**), pre-perinucleolar oocytes (**d**), early and late perinucleolar oocytes (**e**), yolk vesicle oocytes (**e**) and secondary (**g**) and tertiary (**h**) yolk vesicle oocytes. Oogonia (**f**) and somatic cells (**inset f**) only proliferated during testicular involution. Some atretic follicles were observed in the ovarian of males at the beginning of the third RC (**i**). Scale bar = 100 μm (**a,h**), 50 μm (**g**), 25 μm (**e,f,i, inset f**) and 10 μm (**b-d**). OL, ovarian lamellae; OC, ovarian cavity; EPO, early perinucleolar oocytes; LPO, late perinucleolar oocytes; YVO, yolk vesicles oocytes; (arrow heads), proliferative cells; (asterisk), ovarian somatic cells; (white arrows), epithelium lines the ovarian lamellae; (white arrows heads), follicular epithelial cell layer.

The ovary was formed by folds of the germinal epithelium, named ovigerous lamellae that surrounded an ovarian cavity (Fig. 3a). These ovigerous lamellae contained the different types of germ cells embedded in a smooth connective tissue and delimited by epithelial cells (Fig. 3b). Interestingly, the testicular and ovarian areas of the gonad developed independently. Thus, the ovarian area from October to March was composed of nests of oogonia and immature oocytes (pre-perinucleolar and perinucleolar), while the testicular area was developing through its spermatogenesis, spawning and post-spawning stages. The ovarian area started to develop with an asynchronous pattern at the end of March, coinciding with the testicular post-spawning stage, when vitellogenic oocytes in the yolk vesicle stage (also called cortical alveoli stage) were observed. Thus, numerous vitellogenic oocytes in the yolk vesicle stage were observed in April coinciding with the testicular involution stage (April-July). In order to define the germinal cell populations, the morphology and the nuclear and cell diameters of the cells were taken into account (see Table 2 and Fig. 3c, 3d, 3e). In the ovarian area, non-apoptotic cells were observed, while scarce oogonia (Fig. 3f) and some somatic cells (Fig. 3f inset) proliferated, coinciding with the testicular involution stage of the testicular area.

**Table 2:** Feature of the ovarian area of the gonad of gilthead seabream during the second reproductive cycle.

Months	Types of cells present in the ovary	Cell diameter (μm)	Nuclear diameter (μm)	Cytoplasm	Nucleus	Follicle related structures
October-March	Oogonia	16.2 ± 0.3	9.3 ± 0.2	Slightly basophilic	A nucleolus	

	Pre-perinucleolar oocytes	35.0 ± 0.8	18.0 ± 0.4	Highly basophilic	Two or three centrally located nucleoli	
	
April-July	Early perinucleolar oocytes	57.6 ± 1.0	29.6 ± 0.6	Highly basophilic	Numerous nucleoli	
	
	Late perinucleolar oocytes	84.1 ± 3.3	46.9 ± 2.0	Highly basophilic	Numerous nucleoli close to the nuclear envelope	Granulosa
	
	Vitellogenic oocytes at yolk vesicle stage (cortical alveoli stage) *	96.2 ± 2.6	51.0 ± 2.1	Weak basophilic Granules randomly distributed	Numerous nucleoli close to the nuclear envelope	Granulosa Zone radiate

* In April-July also appeared the germ cell types present in the ovary in October-March

### Gonadal development at the end of the second/beginning of the third reproductive cycles

From September to October, the fish have a gonad with both testicular and ovarian areas, which do not undergo further development compared with the same areas described during testicular involution. However, due to the degenerative process that the testicular area underwent during testicular involution, the ovarian area represented 98% of the total gonad. From November onwards, the gametogenic activity restarted and the third RC began, allowing the distinction between both sexes, depending on which area progressed throughout the gametogenesis process. At this time 40% of the population was female and 60% males despite the homogeneous involution of the testicular area observed in the population at the end of the second RC described above. Females showed a more developed ovarian area with vitellogenic oocytes in the secondary yolk vesicle and tertiary yolk vesicle stages (see Table 3 and Fig. 3g,h).

**Table 3:** Feature of the ovarian area of the gonad of gilthead seabream at the end of the second reproductive cycle/beginning of the third reproductive cycle.

Months	Types of cells present in the ovary	Cell diameter (μm)	Nuclear diameter (μm)	Cytoplasm	Nucleus	Follicle related structures
October	No further development					

	Vitellogenic oocytes at secondary yolk vesicle stage *	192.8 ± 9.5	89.0 ± 4.5	Acidophilic globules distributed at the periphery Lipid dropped close to the nucleus		Granulosa Zone radiate Theca cell layer
	
November	Vitellogenic oocytes at tertiary yolk vesicle stage *	310.6 ± 24.9	114 ± 3.5	Numerous eosinophilic globules		Granulosa Zone radiate Theca cell layer
	
	Atretic follicles **			Irregular in shape Numerous acidophilic and some basophilic granules	Highly condensed and basophilic	Flattened cell monolayer

*In November also appeared the germ cell types present in the ovary in October-July. These specimens are females.

**In November, the most developed vitellogenic oocytes undergoes an atretic process. These specimens are males.

Interestingly, the ovarian area of the fish developing as males contained numerous atretic follicles, while the most developed oocytes were vitellogenic oocytes at the yolk vesicle stage. The atretic follicles were formed by a degenerated oocyte surrounded by a flattened cell monolayer (see Table 3 and Fig. 3i).

### Parameters related with the development of the gonad

**Figure 4:**
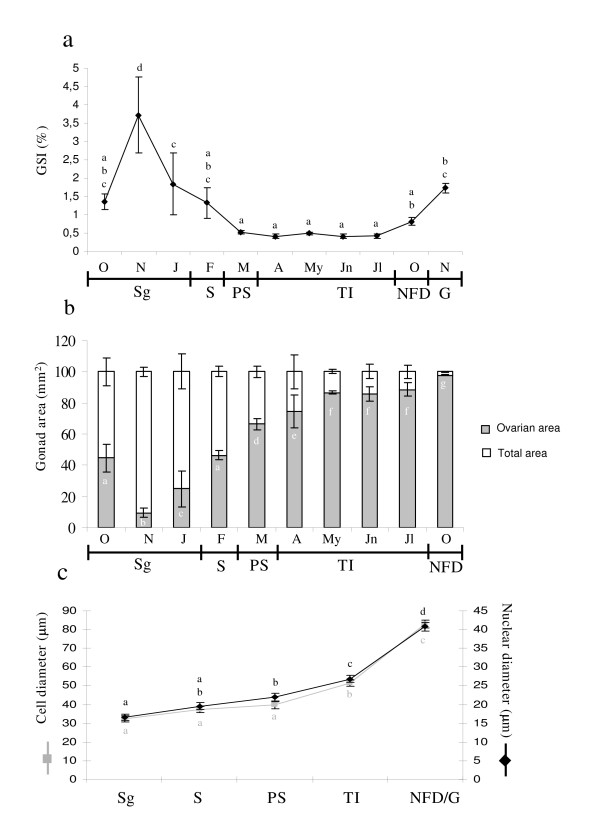
**Parameters related with the development of the gonad**. GSI (**a**), the ratio between the ovarian area (gray part of the bars) and the total area of the gonad (full bars) (**b**) and the cell and nuclear diameters of the oocytes throughout the second RC and at the beginning of the third (**c**). Data represent means ± SEM n = 10–18 fish/month (**a**), n = 3 fish/month (**b**) and n = 111–269 cell/stage (**c**). Different letters denote statistically significant differences between the groups according to a Waller-Duncan test. Sg, Spermatogenesis; S, spawning; PS, post-spawning; TI, testicular involution; NFD, no further development; G, gametogenesis.

As an index of the functional reproductive stage we measured the GSI, variations in which correlated very well with the development of the testicular area in gilthead seabream males (see Table 4 and Fig. 4a). Thus, the GSI increased during spermatogenesis, while in the spawning stage, the shedding of spermatozoa resulted in a sharp decrease in the index, which continued to decrease until the end of the post-spawning stage. In the testicular involution stage the GSI showed little variations. The gonad growth resumed at the beginning of the third RC (November).

**Table 4:** Parameters related with the development of the gonad of gilthead seabream during the second reproductive cycle.

Months	Testicular stages	Ovarian stages	GSI (%)	Ovarian ratio (%)	Ovarian cell diameter means (μm)	Ovarian nuclear diameter means (μm)	Acidophilic granulocytes (%)
October- January	Spermatogenesis	Immature	From 1.4 ± 0.2 to 3.7 ± 1.0 and then to 1.8 ± 0.8	From 44.55 ± 15.06 to 9.52 ± 5.11	32.2 ± 1.7	16.62 ± 0.90	No detected

February	Spawning	Immature	1.3 ± 0.4	46.45 ± 3.26	37.3 ± 1.8	19.54 ± 0.91	4.79 ± 0.96

March	Post-spawning	Immature	0.52 ± 0.04	66.26 ± 3.71	39.6 ± 1.8	21.93 ± 0.99	1.12 ± 0.24

April-July	Testicular involution	Growth: vitellogenic oocytes in yolk vesicles stage	0.41 ± 0.05	From 74.43 ± 10.78 to 88.50 ± 4.20	51.5 ± 1.8	26.82 ± 0.98	From 8.76 ± 1.24 to 3.69 ± 0.67 reaching the maximum value in may (9.25 ± 1.23)

The ovarian ratio (Fig. 4b) and the means of the cell and nuclear diameters of the oocytes (Fig. 4c) were calculated as an index of ovarian development. The ovarian ratio showed great variations throughout the second RC (see Table 4). From October to January the ratio decreased sharply, coinciding with the progression of spermatogenesis in the testicular area of the gonad. However, during post-spawning and testicular involution, the ovarian ratio increased gradually and then stabilized and reached its maximum value at the end of the second/beginning of the third RC (see Table 5 and Fig. 4b). As a result of the development of oocyte populations, mean cell and nuclear sizes increased gradually during the second RC. At the beginning of the third RC both cell and nuclear diameters experienced a great increase (see Tables 4, 5 and Fig. 4c).

**Table 5:** Parameters related with the development of the gonad of gilthead seabream at end of the second reproductive cycle/beginning of the third reproductive cycle.

Months	Testicular stages	Ovarian stages	GSI (%)	Ovarian ratio (%)	Ovarian cell diameter means (μm)	Ovarian nuclear diameter means (μm)	Acidophilic granulocytes (%)
October	No further development		0.82 ± 0.10	97.9 ± 0.4	58.6 ± 2.0	28.2 ± 1.1	3.1 ± 0.7

	No further development	Growth: vitellogenic oocytes at secondary yolk vesicle and tertiary yolk vesicle stages			101.2 ± 5.7	51.2 ± 2.4	
November			1.72 ± 0.13				
	Spermatogenesis	Atretic follicles Oogonia Pre-perinuclear oocytes Early and late perinuclear oocytes Vitellogenic oocytes at yolk vesicle stage					

### DMRT1 gene expression in the gonad

**Figure 5:**
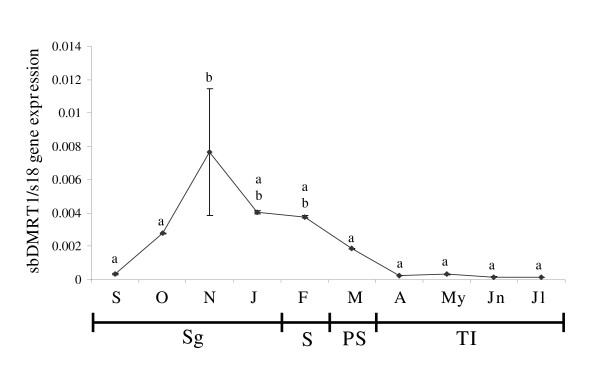
**DMRT1 gene expression in the gonad**. The mRNA levels of DMRT1 were studied by real-time RT-PCR in the gonad at the indicated month. Data represent means ± SEM of triplicate samples. Total mRNA were obtained after mixing the same amount of mRNA from 4–5 fish/month. Different letters denote statistically significant differences between the groups according to a Student-Newman-Keuls test. Sg, spermatogenesis; S, spawning; PS, post-spawning; TI, testicular involution.

In order to determine when the sex change process started, the mRNA level of DMRT1, a gene known to be related with the maintenance of testicular tissue, was measured by real-time RT-PCR. The DMRT1 mRNA levels increased as spermatogenesis proceeded and reached their highest level at the end of the spermatogenesis stage. The level remained steady during spawning and sharply decreased during post-spawning. The DMRT1 mRNA levels were very low during testicular involution (Fig. 5).

### Leukocytes present in the gonad

**Figure 6:**
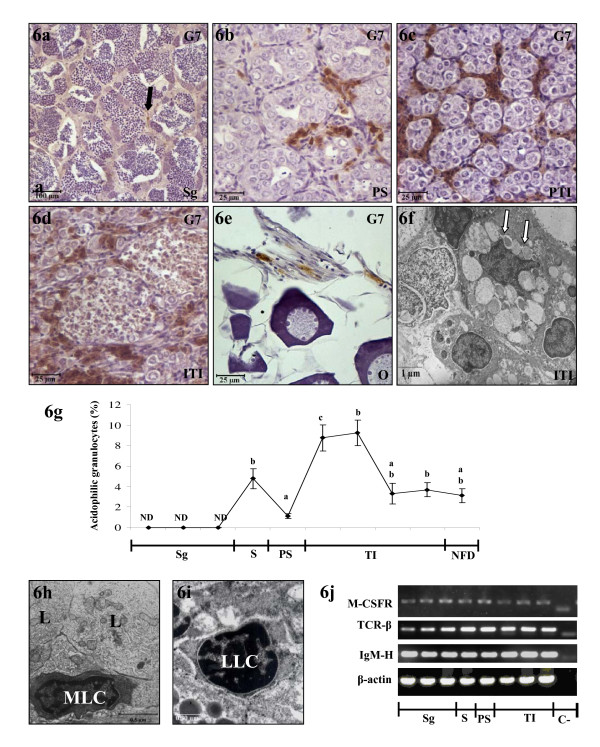
**Leukocytes present in the gonad**. The testicular and ovarian areas of the gonad at different stages of the second RC immunostained with G7 (**a-e**), the ultrastructure of testicular acidophilic granulocytes (**f**), the percentage of gonad acidophilic granulocytes (**g**), the ultrastructure of testicular macrophage-like cells **(h) **and lymphocyte-like cells **(i) **and RT-PCR analysis of M-CSFR, TCR-β and IgM-H genes, as appropriate markers of macrophages and T and B lymphocytes, respectively (**j**). The acidophilic granulocytes appeared in the interstitial tissue of the testis at spermatogenesis (**a**), post-spawning (**b**) and testicular involution (**c,d**). Note that they also appeared between the germ cells at post-spawning (**b**) and around the necrotic areas at testicular involution (**d**). Scattered acidophilic granulocytes were also observed in the interstitial tissue of the ovarian area (**e**). Heterogeneous granules fused to each other were observed in the acidophilic granulocytes closed to the testicular necrotic areas (**f**). Testicular cell suspensions (n = 3–5 fish/month) were immunostained with the G7 and then analyzed by flow cytometry (**g**). The macrophage-like cells in the interstitial tissue in the vicinity of Leydig cells at spermatogenesis stage (**h**). Lymphocyte-like cells in the interstitial tissue (**i**). Total mRNA was obtained to mix the same amount of each mRNA from 4–5 fish/sample (**j**). Scale bar = 100 μm (**a**), 25 μm (**b-e**), 1 μm (**f**), 0.5 μm (**h**), 0.3 μm(**i**). Different letters denote statistically significant differences between the groups according to a Waller-Duncan test. (arrow), G7 positive cells; (white arrows), granules fused to each other; MLC, macrophage-like cell; LLC, lymphocyte-like cell; L, Leydig cell; Sg, Spermatogenesis; S, spawning; PS, post-spawning; TI, testicular involution; NFD, no further development; PTI, peripheral testicular area in the involution stage; ITI, internal testicular area in the involution stage; O, ovary; G, gametogenesis and C-, negative control.

Throughout the second RC, the acidophilic granulocytes (G7 positive cells) were present in the interstitial tissue of testicular (Fig. 6a–d) and ovarian areas (Fig. 6e) and in the connective tissue that limited both areas, surrounding the efferent duct and forming the tunica albuginea. The acidophilic granulocytes infiltrated the gonad in variable numbers (Fig. 6g) and were located in different compartments of the gonad depending on the stage of the RC. The localization of acidophilic granulocytes during spermatogenesis, spawning and post-spawning during the second RC (Fig. 6a,b) coincided with that observed during the first [16]. During testicular involution (Fig. 6c,d) the acidophilic granulocytes were observed in higher numbers in the interstitial tissue of the testicular peripheral area (Fig. 6c) and around the necrotic areas in the testicular internal area (Fig. 6d). Moreover, the granules of the acidophilic granulocytes that surrounded the necrotic areas were heterogeneous in size and electrondensity. Some granules located close to the plasma membrane were beginning to fuse with each other (Fig. 6f).

During spermatogenesis the amount of acidophilic granulocytes was below the limit of detection and increased during spawning. Although the percentage of acidophilic granulocytes rapidly decreased at the end of post-spawning, they increased again during testicular involution to reach maximum numbers in the gonad. This percentage decreased at the end of the testicular involution stage and remained steady until the beginning of the next RC (see Tables 4, 5 and Fig. 6g).

Due to the lack of specific antibodies for macrophages and lymphocytes in the gilthead seabream, we analyzed the presence of these cell types by electron microscopy (Fig. 6h, i) and from the expression of M-CSFR, TCR-β and IgM-H genes in the gonad (Fig. 6j) which were specific markers for macrophages and T and B lymphocytes, respectively. The results showed that macrophage- and lymphocyte-like cells were located in the interstitial tissue of the testis during spermatogenesis. Macrophage-like cells were characterized as irregular cells with polymorphous nuclei and an electron-dense cytoplasm with numerous mitochondria and appeared in close contact with Leydig cell clusters (Fig. 6h). Lymphocyte-like cells appeared as round cells with a large and heterochromatinic nucleus (Fig. 6i). These morphological observations were confirmed by RT-PCR, since the mRNA levels of M-CSFR, TCR-β and IgM-H were found in all stages of the second RC (Fig. 6j).

## Discussion

Our data showed that gilthead seabream, in the western Mediterranean area, developed as males during the first two RCs, while from the third RC onwards the population divided into males and females. This behavior has also been described in studies performed in other Mediterranean regions and indoors with simulated natural photoperiod and temperatures ranging from 15°C to 23°C [9,21]. However, our data are innovative since this is the first time that the cell renewal (proliferation, apoptosis and necrosis) process involved in testicular and ovarian development has been correlated with the leukocyte types present in the gonad. Moreover, the proliferative and apoptotic processes involved in the second RC of the gilthead seabream show interesting differences compared from the first RC [16]. In both cycles spermatogenesis, spawning and post-spawning stages show similar features. However, the last stages of each cycle (resting and testicular involution, respectively) were seen to differ completely. Thus, compared with what happened in post-spawning, the resting stage was characterized by an increase in the number of proliferative cells and no apoptotic cells [16], while during the testicular involution stage, the number of proliferative cells was similar and the number of apoptotic cells increased as did the size of the necrotic areas. In contrast, in the second RC, the degenerative process initiated at post-spawning, was enhanced in the testicular involution stage, resulting in a progressive increase in the ovarian index, which reached 98% of the total gonad at the end of the second RC. Unlike in the first RC, as the testicular area degenerates, the immature oocytes develop and the first vitellogenic oocytes appear. However, the number of proliferative oogonia and ovarian somatic cells in the second RC do not differ from the normal proliferative activity described during each resting stage of the male phase in several sparid species, including the gilthead seabream [22,16]. Despite what has been said before [9], our data demonstrated that during the last stage of each cycle the gonad does not remain latent since cell proliferation and apoptosis allow tissue to be renewed and the beginning of sex change in the first and second RC, respectively.

In seasonal breeding mammals, apoptosis occurs throughout the RC and is related with the amount of spermatogonia and spermatocytes present in the testis rather than being related with seasonal testicular involution [23,24]. However, in the gilthead seabream, apoptosis occurs during post-spawning in the first RC [16] and during post-spawning and testicular involution stages in the second, but not during spermatogenesis as occurs in others species [25,26]. Thus, our data and the data obtained in several fish species demonstrate that germ cell apoptosis and necrotic areas are involved in testicular involution [15,16,27-30].

One important observation of the study is that at the end of the second RC the whole seabream population undergoes a testicular regression process probably triggered by a down-regulation of the expression of genes involved in testicular maintenance. Different genes from a family of genes encoding proteins that contain a DNA-binding motif, called a DM domain, have recently been cloned from a wide range of vertebrates including fish, and these genes have been found to be expressed in the developing gonads and in the adult ovary and/or the testis [8,31-33]. In fact, one DM domain-containing gene, DMRT1 (DM-related transcription factor 1) appears to be involved in a sex-determining cascade and also in testis maintenance [8]. Our data show that the DMRT1 is related with testis development in adults since DMRT1 mRNA levels increase as spermatogenesis proceeds, slightly decreases at the end of the stage and keeps steady during spawning. Interestingly, when testicular involution starts at post-spawning, the mean levels of DMRT1 decrease and reach their minimum values when this process is enhanced during the testicular involution stage. Moreover, DMRT1 expression in trout is high during mid spermatogenesis and also occurs in the pre-vitellogenic ovary and decreases when it starts to develop [8]. This could explain why, in the gilthead seabream, the vitellogenic oocytes do not appear until down-regulation of this gene is really effective. All this supports the idea that in fish the DMRT1 is related not only with sex determination, but also with testicular functions and immature ovary maintenance. Moreover, the very low DMRT1 mRNA levels at the end of the testicular involution stage would explain the remains of a small testicular area (2% of the total gonad) which would allow 60% of the fish population to block the sex change process at the beginning of the third reproductive cycle. In this case, the testis develops again and the maturing oocytes degenerate, becoming atretic follicles as described previously [13,21]. The lack of discernible sex-determining genes such as Sry gene [6], and the existence of genes whose up- or down-regulation determine the development of one sex or the other, would explain the characteristic of the gonad (ovo-testis) in hermaphroditic sparids and the sexual plasticity of teleosts. However, further studies are needed in order to fully understand the gene regulation of the variable pattern of sex determination in fish.

Several studies have dealt with the gilthead seabream sex change and the corresponding female physiology [13,14], but few studies have followed the male physiology throughout the RC and none have dealt with immune and reproductive system interactions. However, as in mammals, the immune and the reproductive systems interact in a complex manner in the gilthead seabream testis, as our previous data on testicular acidophilic granulocytes suggests [15,17].

As regards the presence of leukocytes in the fish gonad, little is known about their role in the seasonal changes observed in this organ. Our previous data from the first RC showed that acidophilic granulocytes infiltrate the gonad following physiological stimuli produced by testicular cells and display impaired immune functions, although they are the only testicular cells that are able to produce reactive oxygen intermediate (ROIs) and intracellularly accumulate IL-1β [15-18]. Interestingly, their location in the gonad during the first RC is similar to that observed during the second one. However, unlike in the first RC, the number of testicular acidophilic granulocytes peaks twice: (i) at the end of spawning/beginning of post-spawning, and (ii) at the beginning of the testicular involution stage when they reach their highest numbers. This finding supports the idea that testicular acidophilic granulocytes are somehow involved in the degenerative process that occurs during these stages. The morphology of testicular acidophilic granulocytes observed in the testicular involution stage also supports this hypothesis. This is the first time that acidophilic granulocytes have been shown to have a different ultrastructure from that observed in testicular and non-activated acidophilic granulocytes [15,20]. Fusion of the granules was observed close to the plasma membrane of the cell, suggesting that these cells might be actively involved in tissue remodeling during testicular involution.

In fish, only a few morphological studies have described macrophages and lymphocytes in the testis [22,34,35] but no experimental studies on the possible roles of these cells in this organ exist due to the lack of specific markers. In rainbow trout, a few macrophages have been observed during spermatogenesis while, after spawning, they were more numerous and appeared near the Sertoli cells, phagocytosing the non-emitted spermatozoa [28,29]. In mammals, macrophages are considered as essential accessory cells for normal reproductive functioning as they are found abundantly in the reproductive tract of males but are somewhat immunosuppressed compared with other resident macrophage populations [1,2,4]. Moreover, Leydig cells and testicular macrophages are functionally related and ROIs and IL-1β produced by testicular macrophages significantly affect Leydig cell physiology [36]. Lymphocytes are also present in the mammalian testis, and approximately 15% of immune cells in the normal adult testis were shown to be lymphocytes [1,2]. Most of these lymphocytes expressed T cell markers with a predominance of CD8+ T cells, whereas B cells were not detectable [1]. In spite of the relatively small number of lymphocytes, the testicular immune-privilege may be a localized phenomenon affecting T cell activation and maturation events [1].

We used electron microscopy analysis of the gonads and studied the expression of specific gene markers to demonstrate that macrophages and both T and B lymphocytes are present in the gonad of the gilthead seabream throughout the second RC, as has been described in mammals [1,37]. Our data show that both macrophage-like cells and lymphocyte-like cells are present in the interstitial tissue of the testicular area of the gonads. Interestingly, in contrast to acidophilic granulocytes, macrophages appear mostly during spermatogenesis in close relation with Leydig cell clusters. Taking all this into account, we hypothesise that macrophages are involved in spermatogenesis, while acidophilic granulocytes are involved in the testicular involution process. However, further studies are necessary to understand whether these cell types are involved in the development and physiology of the gonad as they are thought to be in mammalian vertebrates [1].

## Conclusions

The gilthead seabream specimens from the western Mediterranean area developed as males during the first two RCs. The whole population underwent a testicular degenerative process at the end of the second RC, which was initiated at post-spawning and enhanced at the testicular involution stage, coinciding with maturation of the ovary. However, only 40% of specimens were females in the third RC. DMRT1 might be related with testicular functions and immature ovary maintenance since its expression sharply decreased during the last two stages of the second RC. Interestingly, innate and adaptive immune cells were present in the gonads of gilthead seabream, strongly suggesting a role in spermatogenesis and/or the testicular degenerative process that occur prior to sex change. In fact, two massive infiltrations of acidophilic granulocytes were observed at post-spawning and testicular involution stages.

## Competing interests

The author(s) declare that they have no competing interests.

## Authors' contributions

SL carried out the sampling, gonad processing, developed the methods and wrote the manuscript. ECP participated in the planning of the experiments and carried out the sampling and gene expression analysis, supervised the reproductive stage, took part in the discussion of the results, and helped write the manuscript. AlGa reared the specimens. VM provided the gene sequences, supervised the gene expression analysis and participated in discussing the results. JM participated in the discussion of the results. AGA devised the study, made the statistical analysis, participated in the discussion of the results, and helped write the manuscript.
